# Research on Phenolic Content and Its Antioxidant Activities in Fermented *Rosa rugosa* ‘Dianhong’ Petals with Brown Sugar

**DOI:** 10.3390/antiox13050607

**Published:** 2024-05-15

**Authors:** Yueyue Cai, Merhaba Abla, Lu Gao, Jinsong Wu, Lixin Yang

**Affiliations:** 1 School of Ethnic Medicine, Yunnan Minzu University, Kunming 650504, China; cyy08020602@163.com (Y.C.); gl990@foxmail.com (L.G.); 2 Kunming Institute of Botany, Chinese Academy of Sciences, Kunming 650201, China; maierhaba@mail.kib.ac.cn; 3 Center for Biodiversity and Indigenous Knowledge, Kunming 650034, China

**Keywords:** *Rosa rugosa* ‘Dianhong’, yeast, fermentation, polyphenols, antioxidant activities

## Abstract

Fermented *Rosa rugosa* ‘Dianhong’ petals with brown sugar, a biologically active food popularized in Dali Prefecture, Northwest Yunnan, China, are rich in bioactive compounds, especially polyphenols, exhibiting strong antioxidant activity. This study evaluated their antioxidant activities, total phenolic contents, and concentrations of polyphenols at different fermentation conditions using different assays: DPPH free-radical scavenging activity, Trolox equivalent antioxidant capacity (TEAC), ferric reducing antioxidant power (FRAP), Folin–Ciocalteu assays, and HPLC–MS/MS and HPLC–DAD methods. The results indicated that fermentation significantly increased (*p* < 0.05) the antioxidant activity and polyphenol concentration of *R. rugosa* ‘Dianhong’. Furthermore, *Saccharomyces rouxii* TFR-1 fermentation achieved optimal bioactivity earlier than natural fermentation. Overall, we found that the use of *Saccharomyces rouxii* (TFR-1) is a more effective strategy for the production of polyphenol-rich fermented *R. rugosa* ‘Dianhong’ petals with brown sugar compared to natural fermentation.

## 1. Introduction

*Rosa rugosa* ‘Dianhong’ is well recognized as an edible medicinal plant in Dali Prefecture, NW Yunnan, China. Fermented petals of *R. rugosa* ‘Dianhong’ are a distinctive additive for many traditional foods, such as fermented rose jams, which are traditionally prepared by mixing rose petals with brown sugar and fermenting the mixture in natural fermentation conditions for one year or at least half a year. A previous study found that rose petals exhibit the strongest antioxidant capacity among 12 edible flowers [1]; and possess anti-inflammatory, antibacterial, and anti-diabetic properties [2,3]. Rose buds, hips, and leaves also demonstrated antibacterial, anti-inflammatory, and anticancer activities, which are associated with reduced risk of the general aging process and various age-related diseases [4,5,6,7]. The chemical constituents of rose petals are rich in polyphenols, including gallic acid, quercetin, ellagic acid, kaempferol, rutin, and other compounds [2,8]. Polyphenols in rose petals are considered crucial bioactive components responsible for their antioxidant effects [9]. Due to these qualities, regular consumption of rose-based products is believed to improve health and against the aging process [10,11].

The cultivated area of edible roses is around 6.6 km^2^ in Yunnan, China, with the main cultivars being *R. rugosa* ‘Dianhong’, *R. rugosa* ‘Mohong’, and *R. chinesis* cv. ‘JinBian’ [12]. *R. rugosa* ‘Dianhong’ is the most popular and widely cultivated, and the petals of *R. rugosa* ‘Dianhong’ are primarily used to make fermented rose jams and are occasionally used to make rose wine, rose vinegar, and rose juice and tea. Most Bai people make fermented *R. rugosa* ‘Dianhong’ products by hand from March to May every year and begin consuming them after six months or one year of fermentation. Scientific research may provide more evidence of economic value to support the production of this local food, such as medicinal and nutritional values. Although there are many studies on the chemical constituents and multiple bioactivities of *Rosa rugosa* [13,14], only a few studies on the bioactive compounds and related bioactivities of fermented *R. rugosa* specifically related to fermented *R. rugosa* ‘Dianhong’ products and processes have been reported [15,16].

Previous studies have reported that microbial fermentation was able to enhance the biological activities of plants [17,18]. The ability of fermentation to increase antioxidant activity is primarily attributed to microbial hydrolysis reactions [19]. A recent study showed that using *Lactiplantibacillus plantarum* B7 and *Bacillus subtilis natto* for fermentation increased the polyphenol content in rose residue, leading to improved antioxidant capacity. Fifteen phenolic compounds were quantified in the fermented broth, with notable increases in the concentration of gallic acid and quercetin [20]. It has been reported that harvested plants exhibit an increase in phenolic compound content post-fermentation, which may be attributed to an increase in total phenolics [21,22].

Traditionally, fermented rose in Dali Bai communities is primarily prepared from *R. rugosa* ‘Dianhong’ and *R. rugosa* ‘Mohong’ petals. It is commonly produced through a long period of natural fermentation and the brown sugar content that is most frequently used is 50%. Our previous study used the key strain *Saccharomyces rouxii* (TFR-1), which was isolated from the traditional fermented jam of *R. rugosa* ‘Dianhong’, for fermentation research. It verified that fermented *R. rugosa* ‘Dianhong’ with a lower brown sugar content (25%) exhibited a higher total phenolic content (TPC) and stronger antioxidant and anti-inflammatory activities compared to those with a higher sugar content (50%) [23]. However, scientific research is still limited in systematically investigating the changes in the main phenolic contents and antioxidant activities of naturally fermented *R. rugosa* ‘Dianhong’ methanol extracts (NFR) and yeast (*Saccharomyces rouxii* TFR-1) fermented *R. rugosa* ‘Dianhong’ methanol extracts (YFR) during fermentation. Therefore, it is hypothesized that rose petals fermented with the dominant strain TFR-1 can increase the phenolic contents and antioxidant activities earlier in the fermentation process compared to natural fermentation. Furthermore, traditional fermented rose craftsmanship is characterized by limited-scale craftsmanship, lack of quality standards, and long fermentation periods, as well as a high cost, which need to be improved in further research. Therefore, the current study aimed to determine the changes in total phenolic content (TPC), DPPH, ABTS^+^ scavenging, ferric reducing antioxidant potential (FRAP), and superoxide dismutase (SOD) activity, as well as the content of phenolic substances in NFR and YFR during fermentation. High-performance liquid chromatography–tandem mass spectrometry (HPLC–MS/MS) was used for the qualitative and quantitative analysis of 15 phenolic compounds in NFR and YFR, which exhibited the highest TPC, SOD, and antioxidant activity. Moreover, the changes in four major phenolic compounds that significantly (*p* < 0.05) increased post-fermentation were investigated using the HPLC–DAD method.

## 2. Materials and Methods

### 2.1. Materials and Chemicals

#### 2.1.1. Materials

Fresh rose (*Rosa rugosa* ‘Dianhong’) petals were collected in May 2022 from Dali Bai Autonomous Prefecture, Yunnan, China. All voucher specimens were identified by taxonomists at the Kunming Institute of Botany, Chinese Academy of Sciences, and were pre-cooled immediately after collection, and then, stored at −20 °C. *Saccharomyces rouxii* (TFR-1) was acquired from the China General Microbial Culture Center (accession no. CGMCC No. 19335).

#### 2.1.2. Chemicals

Folin–Ciocalteu reagent and gallic acid (GA) were purchased from Beijing Solarbio Science & Technology Co., Ltd. (Beijing, China). 2,2-Diphenyl-1-picrylhydrazyl (DPPH) was purchased from Sigma (Steinhelm, Germany). 2,4,6-Tri(2-pyridyl)-s-triazine (TPTZ) was bought from Sigma-Aldrich (St. Louis, MO, USA). Methanol, formic acid, and 2,2′-azino-bis (3-ethylbenzothiazoline-6-sulfonic acid) (ABTS) were obtained from the Macklin Chemical Factory (Shanghai, China). Ascorbic acid (Vc), sodium carbonate, iron (III) chloride hexahydrate, sodium acetate, hydrochloric acid, potassium persulfate, sodium nitrite, and sodium hydroxide were sourced from Tianjin Chemical Factory (Tianjin, China). Phenolic standards, such as gallic acid (GA), rutin, quercetin, and ellagic acid, were supplied by D&B (Shanghai, China). Syringic acid, apigenin, and 4-hydroxybenzoic acid were purchased from Mreda (Beijing, China). Vanillic acid, kaempferol, naringenin, and quercitrin were bought from the Macklin Chemical Factory (Shanghai, China).

### 2.2. Sample Fermentation and Extraction

#### 2.2.1. Fermentation Sample Preparation

*S. rouxii* (TFR-1) was activated and cultured on YPD solid medium, purified three times, and a single colony was picked and cultured in YPD liquid medium at 28 °C and 120 r/min for 36 h. Fresh *R. rugosa* ‘Dianhong’ petals were mixed with brown sugar in a ratio of 3:1 and kneaded for half an hour. After kneading, 30 g of the mixture was taken out and extracted as unfermented *R. rugosa* ‘Dianhong’ methanol extracts (UFR) on day 0. The remaining mixture was separated into two groups: one for natural fermentation and the other for yeast (TFR-1) fermentation. The natural fermentation mixture was fermented without sterilization or yeast. The yeast (TFR-1) fermentation mixture was sterilized for 2 h using ultraviolet light, and then, inoculated with 2% (*v*/*v*) TFR-1 (1.7 × 10^6^) CFU/mL. Both groups were fermented at 30 °C and 120 r/min in a shaker incubator. Fermented samples (each 30 g) were collected on days 7, 14, 21, 28, and 35. Each sample was extracted using 95% (*v*/*v*) aqueous methanol. The naturally fermented and yeast-fermented *R. rugosa* ‘Dianhong’ methanal extracts were expressed as NFR and YFR, respectively. The extraction procedure of all samples can be seen in Section 2.2.2.

#### 2.2.2. Fermentation Sample Extraction

Each sample (30 g) was extracted twice in 150 mL of 95% (*v*/*v*) aqueous methanol under an ultrasonic action temperature of 25 °C, an ultrasonic power of 500 W, and an ultrasonic time of 40 min. The solutions were filtered and combined, then evaporated under reduced pressure at 45 °C to obtain UFR, NFR, and YFR.

### 2.3. Determination of Total Phenolic Content (TPC)

The total phenolic content was determined using an improved Folin–Ciocalteu method [24]. Folin–Ciocalteu reagent (125 µL) was added to each gallic acid solution (0–10 µg/mL) and mixed thoroughly for 2 min. Then, a 250 µL mix of 12% CaCO_3_ and distilled water was added. The mixture was incubated at 25 °C for 60 min. The absorbance was recorded at 765 nm using an automated enzyme labeler. TPC was expressed as mg of gallic acid equivalent (GAE)/g of powder on dry weight (DW). The standard curve regression equation obtained was y = 0.0924x + 0.0249 (*R*^2^ = 0.9981).

### 2.4. In Vitro Antioxidant Assays

#### 2.4.1. DPPH Radical Scavenging Assay

The antioxidant activities of UFR, NFR, and YFR were evaluated using the ability of DPPH (1,1-diphenyl-2-trinitrophenylhydrazine) to scavenge free radicals in vitro, a method described in Kumari et al. [25] with minor modifications. A volume of 100 µL of *R. rugosa* ‘Dianhong’ methanol extract (0.20 mg/mL) was added to 100 µL of 0.1 mM DPPH methanol solution. The absorbance was recorded at 517 nm using an automated enzyme labeler. The DPPH radical scavenging ability was calculated and expressed using the following equation:

%DPPH scavenging activity = (1 − A_sample_/A_control_) × 100 (1)

Here, A_sample_ is the absorbance of the tested sample, and A_control_ is the absorbance of the control (containing all reagents except the test compound).

#### 2.4.2. ABTS^+^ Radical Scavenging Assay

The ABTS^+^ free-radical scavenging capacity method was applied with minor modifications [26]. An ABTS^+^ solution was prepared by mixing and reacting potassium persulfate (1.4 mmoL/L) and ABTS^+^ (7 mmoL/L) in a 1:1 ratio and allowing the mixture to stand in the dark for 12–16 h before use. The ABTS^+^ solution was diluted in methanol to achieve a value of 0.70 ± 0.02 at 734 nm absorbance. Afterward, 50 µL of each sample (0.05–0.125 mg/mL) was added to 150 µL of the diluted ABTS^+^ solution. After incubation in the dark for 30 min, absorbance was recorded at 734 nm. The ABTS^+^ radical scavenging ability was calculated and expressed as the following equation:

%ABTS^+^ scavenging activity = (1 − A_sample_/A_control_) × 100 (2)

where A_sample_ is the absorbance of the tested sample, and A_control_ is the absorbance of the control (containing all reagents except the test compound).

#### 2.4.3. Ferric Reducing Antioxidant Potential (FRAP) Assay

The antioxidant activities of UFR, NFR, and YFR were evaluated using the FRAP method with slight modifications [27]. Samples (0.5 mL) of *R. rugosa* ‘Dianhong’ methanol extracts and deionized water were prepared in different concentrations in a 5 mL EP tube, then 0.5 mL of phosphate buffer (pH = 6.8) and 0.5 mL of 1% potassium ferricyanide solution were added in turn. The mixtures were bathed at 50 °C for 20 min. Subsequently, 0.5 mL of 10% trichloroacetic acid solution was added, mixed, and then, centrifuged at 3000 r/min for 10 min. A volume of 0.5 mL of the supernatant was transferred to a 2 mL EP tube, to which 0.5 mL of pure water and 0.1 mL of 0.1% ferric chloride were added. After reacting for 5 min at room temperature, the optical density (OD) value was measured at 700 nm. The assay was conducted in triplicate.

#### 2.4.4. Measurement of Superoxide Dismutase (SOD) Activity

SOD, a metal enzyme found in organisms, is an important oxygen radical scavenger that catalyzes the conversion of superoxide anions into H_2_O_2_ and O_2_. SOD is not only a superoxide anion scavenging enzyme but also a major H_2_O_2_ generating enzyme, playing an important role in the biological antioxidant system. Through the xanthine and xanthine oxidase reaction system, superoxide anion (O_2_^−^) is produced, which can reduce nitrogen blue tetrazolium to generate blue formazan. This formazan absorbs at 560 nm. SOD removes O_2_^−^, thus inhibiting the formation of formazan. The darker the color of the reaction solution, the lower the activity of SOD, and vice versa. In this study, the SOD activity of unfermented and fermented *R. rugosa* ‘Dianhong’ methanol extracts (UFR, NFR, and YFR) were determined according to the test kit (D799593, Sangon Biotech Co., Ltd., Shanghai, China) used and described in Dai et al. [28]. To measure SOD activity, 90 µL of extract dilution (1 mg/mL) was centrifuged at 4200 r/min for 10 min, and the supernatant was collected on ice to be measured. The remaining experimental steps were conducted in accordance with the instructions provided with the kit. Once all the reagents were fully mixed, the mixture was heated in a water bath for 30 min at 37 °C. The OD value was measured at 560 nm. Three replicates were measured. The formula for the SOD enzyme activity of the extract is as follows:

SOD enzyme activity (U/mg) = 11.1 × (Percentage inhibition/(1 − Percentage inhibition)/W × F

where W is the weight of sample, and F is the sample dilution factor.

### 2.5. Phenolic Compounds Content Analysis

#### 2.5.1. Targeted Metabolomics Analysis

In this study, targeted metabolomics analysis of polyphenols was conducted using HPLC–MS/MS with a 6500 QTRAP triple quadrupole mass spectrometer (Framingham, MA, USA) coupled to an ExionLC system (Applied Biosystems/Sciex). MS analyses were carried out using electrospray ionization (ESI) in both positive- and negative-ion modes, employing multiple reaction monitoring (MRM) scans. The source temperature was set at 450 °C. Specific transitions (*m*/*z*) and retention times (RTs) facilitated the identification and quantification of polyphenols, detailed in Table 1. Chromatographic separation occurred on a Phenomenex Gemini^®^ 3 µm NX-C18 100 Å LC column (50 × 2 mm), maintained at 40 °C. The mobile phase consisted of 0.1% aqueous formic acid (A) and equivalent proportions of methanol and acetonitrile solution (B) at a flow rate of 0.3 mL/min. Gradient elution was set as follows: 0–1.5 min, 15% B; 1.5–5.0 min, 15–100.0% B; 5.0–6.5 min, 100% B; 6.5–7.5 min, 100–15% B; 7.5–9.5 min, 15% B. The sample injection volume was 1 µL, with compounds identified and quantified using MultiQuant 3.0.2 against reference standards.

#### 2.5.2. HPLC–DAD Analysis of Phenolic Compositions

Fermented extracts were characterized for phenolic compounds using HPLC–UV/DAD analyses, as described by Abdellatif et al. [29]. The analysis was carried out on column C18 (250 × 4.5 mm, 5 μm) with a flow rate of 1.0 mL/min and column temperature set to 35 °C. The injection volume was 10 μL. The chromatographic conditions employed solvent C (methanol, HPLC grade) and solvent D (0.3 (*v*/*v*) aqueous formic acid), with the following gradient: 0 min: 10% C + 90% D; 10 min: 20% C + 80% D; 25–30 min: 25% C + 75% D; 35–60 min: 35% C + 65% D; 65–75 min: 50% C + 50% D; 80 min: 80% C + 20% D; 85 min: 90% C + 10%D; ending at 90 min with 100% C. Detection was effected at 280 nm. The phenolic compounds in the extracts were identified by comparing their retention times and the UV spectra obtained with those of known standards. The external standard technique was used to quantify phenolics by peak integration.

### 2.6. Statistical Analysis

During the analysis, all results were expressed as the mean ± standard deviation (SD) of three independent determinations (n = 3), and differences at *p* < 0.05 were considered statistically significant. SPSS Statistics 20.0 (IBM, Armonk, NY, USA) was used to perform one-way and two-way analysis of variance (ANOVA) with Duncan’s multiple range test. Correlation analyses were performed using Pearson analysis to assess the relationship between phenolic content and antioxidant activities.

## 3. Results and Discussion

### 3.1. TPC Values of Unfermented and Fermented R. rugosa ‘Dianhong’

**Figure 1:**
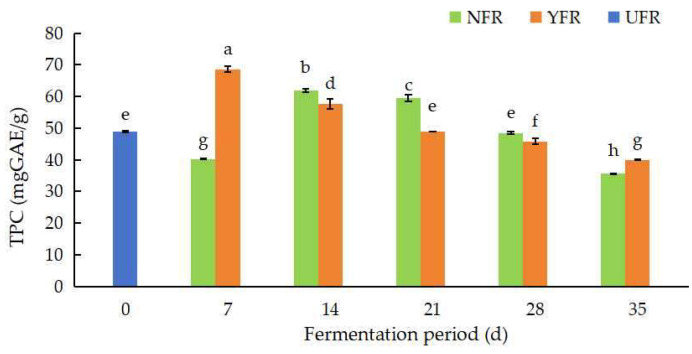
Changes in TPC values of *Rosa rugosa* ‘Dianhong’ extracts before and after fermentation at different fermentation periods. Values are presented as mean ± standard deviation (n = 3) and evaluated by two-way ANOVA (post hoc test: Duncan test). Different lowercase letters (a–h) on the bars indicate significant differences (*p* < 0.05) in TPC values among *R. rugosa* ‘Dianhong’ extracts, while the same lowercase letters (a–h) indicate no significant difference (*p* > 0.05).

The TPC values of the *R. rugosa* ‘Dianhong’ sample are shown in Figure 1. The TPC values varied from 35.54 ± 0.24 to 68.58 ± 1.02 mg GAE/g. The TPC values of NFR and YFR were significantly different (*p* < 0.05) at different fermentation periods. The TPC values of NFR (7–14 d) and YFR (14–21 d) were significantly higher (*p* < 0.05) than the UFR (0 d). The TPC value of NFR increased significantly (*p* < 0.05) from 7 d to 14 d, peaking at 61.85 mg GAE/g at 14 d of fermentation. After 14 d, the TPC value of NFR showed a significantly decreasing (*p* < 0.05) trend. The TPC value of YFR increased at the initial stage of fermentation, and then, showed a decreasing trend thereafter. The highest TPC value (68.58 mg GAE/g) occurred in YFR at 7 d of fermentation and was significantly higher (*p* < 0.05) than the highest TPC value (61.85 mg GAE/g) in NFR. Compared to natural fermentation (NFR), the fermentation period to reach the maximum TPC can be shortened by using *S. rouxii*, likely due to microbial modification of bioactive compounds (such as phenolic compounds and flavonoids), enhancing TPC in the fermented *R. rugosa* ‘Dianhong’ petals with brown sugar [30,31]. In brief, fermentation markedly increased (*p* < 0.05) the TPC values of fermented *R. rugosa* ‘Dianhong’, with YFR (7 d) exhibiting a higher TPC value than NFR (14 d) and significantly surpassing the UFR (0 d). Comparable findings were reported for rose jams (containing 75% white sugar) fermented with *Pediococcus pentosaceus* MP13, showing increased TPC after 7 d of fermentation [32]. Therefore, fermentation with strains possessing excellent fermentation properties could markedly increase the TPC values of rose jams over natural fermentation, contributing to the standardization and quality control of fermented rose products.

### 3.2. In Vitro Antioxidant Assays

#### 3.2.1. The DPPH, ABTS^+^, and FRAP Radical Scavenging Assay

**Figure 2:**
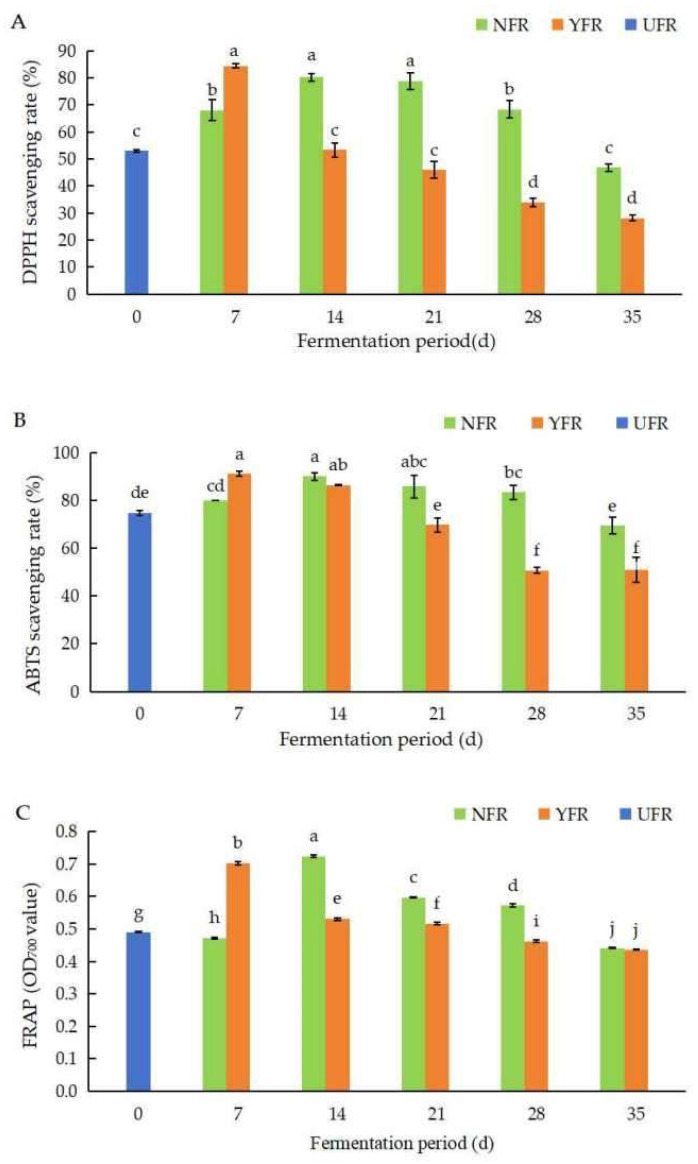
Changes in DPPH, ABTS^+^, and FRAP values of unfermented and fermented *Rosa rugosa* ‘Dianhong’ extracts. (**A**) The DPPH values of *R. rugosa* ‘Dianhong’ extracts; (**B**) the ABTS^+^ values of *R. rugosa* ‘Dianhong’ extracts; (**C**) the FRAP values of *R. rugosa* ‘Dianhong’ extracts. Values are presented as mean ± standard deviation (n = 3) and evaluated by two-way ANOVA (post hoc test: Duncan test). Different lowercase letters (a–j) on the bars indicate that *R. rugosa* ‘Dianhong’ extracts are significantly different (*p* < 0.05), while the same letters lowercase letters (a–j) indicate no significant differences (*p* > 0.05).

*R. rugosa* petals have demonstrated strong DPPH and ABTS^+^ radical scavenging capacity, obtaining the highest FRAP values among 12 edible flowers [11]. Microbial fermentation can enhance the antioxidant activities of ferments, and the fermentation time significantly affects the antioxidant activities of the fermentation product [33]. This study focuses on a systematic evaluation of the antioxidant activity of fermented *R. rugosa* ‘Dianhong’ (FRD) with less brown sugar content (25%) and elevated antioxidant activity. It also compares the traditional spontaneously prepared rose jams (NFR) with improved yeast-based fermented rose jams (YFR). The changes in DPPH, ABTS^+^, and FRAP values of FRD, fermented through natural and yeast-based processes at varying fermentation times, were investigated (Figure 2).

The DPPH values of *R. rugosa* ‘Dianhong’ extracts are shown in Figure 2A. Generally, the DPPH values of NFR and YFR initially increased with fermentation time, peaking at 14 d and 7 d, respectively, before declining. Notably, YFR fermented for 7 d exhibited the highest DPPH value (84.44%), reaching optimal fermentation 7 days earlier than NFR. This represented a significant enhancement of 31.46% (*p* < 0.05) compared to the unfermented UFR (0 d).

The ABTS^+^ values of *R. rugosa* ‘Dianhong’ extracts are shown in Figure 2B. Overall, NFR and YFR exhibited notable ABTS^+^ radical scavenging activity (50.71–91.01%). The ABTS^+^ values for both NFR and YFR increased during the initial stage of fermentation (7–14 d), and then, significantly decreased (*p* < 0.05) as fermentation time extended. The peak ABTS^+^ values for NFR (89.90%) and YFR (91.01%) were obtained at 14 d and 7 d of fermentation, respectively. These values were significantly higher (*p* < 0.05), by 15.14% and 16.25%, respectively, compared with UFR (0 d).

The FRAP values of *R. rugosa* ‘Dianhong’ extracts are shown in Figure 2C. The trends in FRAP values for NFR and YFR paralleled those of the ABTS^+^ values (Figure 2B,C), initially increasing with fermentation time before subsequently decreasing. The FRAP values for NFR (14–28 d) and YFR (7–21 d) were significantly higher (*p* < 0.05) than for UFR (0 d). Both NFR and YFR obtained their highest iron reducing capacities at 14 d and 7 d of fermentation, respectively, indicating that fermentation for 7 d is the optimal fermentation period for YFR to reduce fermentation time while enhancing antioxidant capacity.

In summary, fermenting *R. rugosa* ‘Dianhong’ for 7 days significantly enhances the DPPH, ABTS^+^, and FRAP values of YFR, with YFR reaching peak values faster than NFR. This trend aligns with other findings, where, for instance, the antioxidant activity of kombucha showed an increase followed by a decrease during the fermentation process [21]. During the pre-fermentation period, antioxidant active substances such as TPC dissolve due to the action of hydrolytic enzymes. Studies have indicated that microbial fermentation can increase the content of polyphenolic compounds in rose petals, thereby boosting their antioxidant capacity [16]. In the later stages of fermentation (beyond seven days), the growth of *S. rouxii* TFR-1 is constrained by limited space and nutrients, leading to reduced fermentation activity and a subsequent decrease in the ability to scavenge free radicals as the fermentation period extends.

#### 3.2.2. SOD Activity

**Figure 3:**
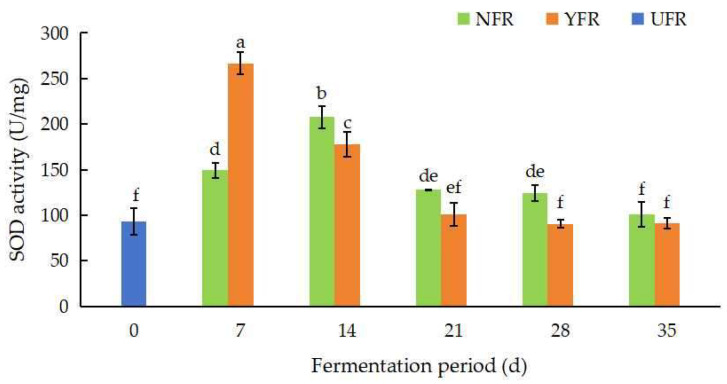
Changes in SOD activity of *Rosa rugosa* ‘Dianhong’ extracts. Values are presented as mean ± standard deviation (n = 3) and evaluated by two-way ANOVA (post hoc test: Duncan test). Different lowercase letters (a–f) on the bars indicate that *R. rugosa* ‘Dianhong’ extracts are significantly different (*p* < 0.05), while the same lowercase letters (a–f) indicate no significant differences (*p* > 0.05).

Superoxide dismutase (SOD) enzymes, which remove free radicals through disproportionation reactions, provide an indirect measure of the antioxidant activity of plants. The SOD activity of unfermented and fermented *R. rugosa* ‘Dianhong’ extracts over different fermentation periods is shown in Figure 3. Microorganisms, including *S. rouxii*, are known to produce SOD [34], which explains the observed increase in SOD activity in the fermented extracts. Espirito-Santo et al. [35] also reported that the SOD activity in apple juices significantly increased by 37.11% due to fermentation with *Lactiplantibacillus* strains. In this study, the SOD activity of NFR (7–14 d) and YFR (7 d) showed a significant increase at the initial stage of fermentation (Figure 3), which was related to the changes in polyphenol content [36]. NFR’s SOD activity peaked (207.65 U/mg) at 14 d of fermentation, then decreased. Remarkably, YFR reached its highest SOD activity at 7 d of fermentation, indicating that YFR achieves optimal fermentation (7 d) sooner than NFR (14 d). In addition, at 7 d, YFR’s SOD activity was significantly higher (by 1.87 times; *p* < 0.05) than UFR (0 d).

### 3.3. Compound Detection

#### 3.3.1. The Analysis of Phenolic Compositions

Although the polyphenols and antioxidant activity of *R. rugosa* have been reported, the dynamics of polyphenol content in fermented *R. rugosa* ‘Dianhong’ petals with brown sugar remain less explored. Nguela et al. reported that in a partially aerobic environment, grape wine’s polyphenols were released progressively, impacting yeast fermentative capacity [37]. In the present study, using various fermentation techniques, we detected a broader range of major compounds in fermented *R. rugosa* ‘Dianhong’ extracts through HPLC–MS/MS analysis (Table 1).

**Figure 4:**
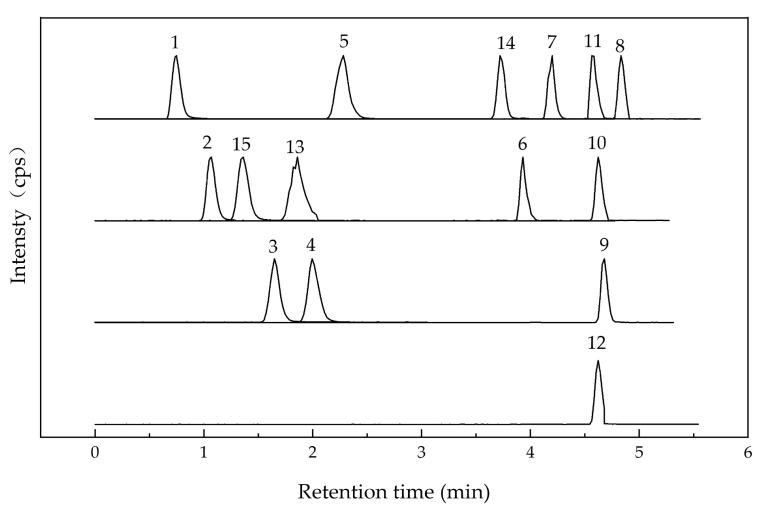
HPLC–MS/MS chromatogram of 15 standard peaks: 1. gallic acid; 2. protocatechuic acid; 3. p-hydroxybenzoic acid; 4. vanillic acid; 5. syringic acid; 6. rutin; 7. quercitrin; 8. quercetin; 9. naringenin; 10. kaempferol; 11. apigenin; 12. luteolin; 13. chlorogenic acid; 14. ferulic acid; 15. catechin.

Polyphenols in UFR (0 d), NFR (14 d), and YFR (7 d) were identified and quantified using a validated high-performance liquid chromatography–tandem mass spectrometry (HPLC–MS/MS) method. Electrospray ionization (ESI) was used for analyses in both positive- and negative-ion modes (Table 1), and chromatograms of standards are shown in Figure 4). A total of 15 phenolic compounds from *R. rugosa* ‘Dianhong’ samples were identified, comprising 7 phenolic acids (gallic acid, protocatechuic acid, p-hydroxybenzoic acid, vanillic acid, syringic acid, chlorogenic acid, and ferulic acid) and 8 flavonoids (rutin, quercitrin, quercetin, naringenin, kaempferol apigenin, luteolin, and catechin).

In Table 1, the concentrations of eight phenolic compounds (gallic acid, *p*-hydroxybenzoic acid, vanillic acid, quercitrin, quercetin, naringenin, kaempferol, apigenin) were significantly increased (*p* < 0.05) in NFR (14 d) and YFR (7 d) compared to the unfermented sample UFR (0 d). In addition, the concentrations of luteolin and ferulic acid in YFR were also significantly increased (*p* < 0.05) compared to UFR (0 d). These compounds may contribute to the significant increase (*p* < 0.05) in TPC, antioxidant, and SOD activity of the fermented samples (NFR (14 d) and YFR (7 d)). The highest concentration (175.02 ± 14.13) μg/g among all samples was of quercitrin in YFR (7 d), followed by (152.97 ± 7.94) μg/g in NFR (14 d), which increased by 32.64% and 15.93%, respectively, compared to the UFR (0 d) (131.95 ± 7.30) μg/g. Remarkably, the content of quercetin increased by 3.62 times in NFR (14 d) and 3.74 times in YFR (7 d), respectively, compared to the UFR (0 d).

Briefly, the total content of tested phenolic compounds, including gallic acid, vanillic acid, syringic acid, apigenin, luteolin, and ferulic acid in YFR (yeast-fermented), were significantly higher (*p* < 0.05) than in NFR (naturally fermented) and UFR (unfermented). Furthermore, eight phenolic compounds significantly increased (*p* < 0.05) in NFR (14 d) and YFR (7 d) compared to UFR (0 d). These increases paralleled the rises in TPC, DPPH, ABTS^+^, FRAP, and SOD values, which indicated that these compounds at least partly contributed to the enhanced antioxidant activity observed in NFR (14 d) and YFR (7 d). Similar results were found in a study on yeast-based fermented Kei apple juice (*Dovyalis caffra* L.), which showed increased levels of phenolic acids, such as ferulic, caffeic, chlorogenic, protocatechuic, and sinapic acids [38].

#### 3.3.2. The Analysis of Phenolic Compositions

**Figure 5:**
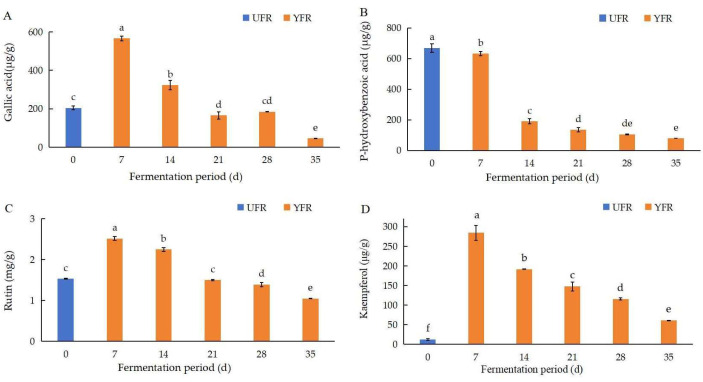
Changes in major phenolic compound contents during the fermentation of *Rosa rugosa* ‘Dianhong’ extracts for different fermentation periods: (**A**) gallic acid; (**B**) p-hydroxybenzoic acid; (**C**) rutin; (**D**) kaempferol. Values are presented as mean ± standard deviation (n = 3) and evaluated by one-way ANOVA (post hoc test: Duncan test). Different lowercase letters (a–f) on the bars for the same compound represent significant differences (*p* < 0.05), while the same lowercase letters (a–f) for the same compound represent no significant differences (*p* > 0.05).

**Figure 6:**
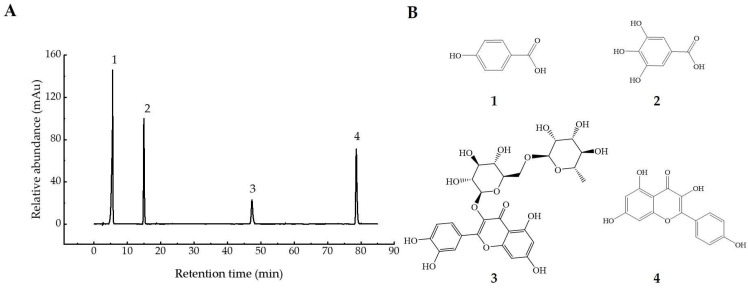
Information on the four phytochemical standards. (**A**) HPLC chromatograms of 0.1 mg/mL mixed standards recorded at 280 nm. (**B**) the chemical structures of the four standards: (**1**) gallic acid; (**2**) p-hydroxybenzoic acid; (**3**) rutin; and (**4**) kaempferol.

The changes in four phenolic compounds (gallic acid, p-hydroxybenzoic acid, rutin, kaempferol) in YFR during fermentation were identified and quantified using the previously mentioned HPLC–DAD method (Figure 5). The chromatograms and structures of the four quantified compounds in YFR are shown in Figure 6. As the major phenolic compounds found in YFR, high levels of these four compounds also contribute to the bioactivity of YFR and the UFR (0 d). These four compounds were detected in all YFR and UFR (0 d) samples. A new method for the quantification of these four components in YFR was successfully developed, using calibration curves of standards fitted by linear regression analysis. The linear regression analysis was set up by plotting peak area (y) against the content (x, mg) of the standard solution, described as follows: y = 259.89x + 41.642, R^2^ = 0.9995 (for gallic acid; the linear range is 0.2–2.0 μg/g); y = 156.8x + 25.177, R^2^ = 0.9995 (for p-hydroxybenzoic acid; the linear range is 0.2–2.0 μg/g); y = 69.028x + 3.3705, R^2^ = 0.9990 (for rutin; the linear range is 0.2–2.0 μg/g); y = 160.71x + 12.53, R^2^ = 0.9995 (for kaempferol; the linear range is 0.2–2.0 μg/g). The external standard method was performed for the simultaneous determination of the content of the four phenolic compounds.

According to the results of the HPLC–DAD test, the concentrations of gallic acid, rutin, and kaempferol were significantly increased (*p* < 0.05) and reached their peak levels after 7 d of fermentation, then significantly decreased with further time extension (Figure 5). At 7 d of fermentation, the concentrations of gallic acid, rutin, and kaempferol were significantly increased (*p* < 0.05), by 1.77, 0.64, and 22.12 times, respectively, compared to the UFR (0 d). Interestingly, the content of kaempferol (35 d) was still significantly (*p* < 0.05) higher than the UFR (0 d), indicating that the yeast fermentation of *R. rugosa* ‘Dianhong’ significantly influenced the changes in kaempferol content during fermentation. The content of p-hydroxybenzoic acid in YFR showed a significant decreasing trend during fermentation and was reduced by 88.13% after 35 d of fermentation. Phenolic and flavonoid compounds exhibit different antioxidant activities based on their structural conformation, the number and arrangement of the hydroxyl groups, and their position in the structure [39]. Generally, phenolic acids and flavonoids are effective hydrogen donors because they contain carboxylic acid groups and hydroxyl functional groups that are easily ionized [40]. Previous research reported that during the fermentation of pickled tea, high levels of gallic acid can accumulate through anaerobic fermentation over an appropriate duration [41]. This process may hydrolyze complex polyphenols into simpler, more bioactive, forms, resulting in enhanced antioxidant activities [42]. Furthermore, other enzymatic reactions such as hydroxylation, oxidation, and reduction may contribute to the production of phenolic compounds with higher antioxidant activities, thereby enhancing the antioxidant properties of ferments during fermentation [43].

### 3.4. Relevance Analysis

A correlation analysis was carried out to investigate the correlation between phenolic content and the bioactivities of YFR further. The analysis showed that the TPC of YFR was highly significantly correlated (*p* < 0.01) with DPPH, ABTS^+^, and FRAP. There was a significant correlation (*p* < 0.05) between SOD and TPC. The four phenolics demonstrated a positive correlation with antioxidant capacity. These findings suggest that phenolic compounds are responsible for the antioxidant activity of YFR. The variation in bioactivities of YFR during fermentation was correlated with the differences in phenolic content. Similar results have been reported in previous studies related to the *Rosa* genus. For example, Liaudanskas et al. reported that the total amount of phenolic compounds in fruit extracts of the *Rosa* genus had a strong correlation with their radical scavenging and reducing activities [6]. Al-Yafeai et al. reported [44] that changes in the antioxidant capacity of rosehips are linked to alterations in their bioactive compound content, indicating that fermentation significantly affects the phenolic compounds, and thus, the bioactivities in YFR. In summary, our results indicate that YFR (7 d), which is fermented with yeast (TFR-1), leads to a product with strong antioxidant activities and a higher concentration of phenolic acids in a shorter time. Microbial fermentation can change the biochemical compounds and bioactivities of fermented food. Fermented foods with antioxidant activity and other bioactivities could benefit consumer health and have great potential for the nutrition, food, and cosmetic industries [32]. Hu et al. reported that after fermentation of rose residue by *Lactiplantibacillus plantarum* and *Bacillus subtilis natto*, the polyphenol content and related activities exhibited an initial increase followed by a decreasing trend during fermentation, which showed similar changing trends to this study [20]. Furthermore, certain bacteria, molds, and yeast can physically and chemically decompose or break down the cell walls and indigestible coatings of these products, thereby promoting the release of nutrients. Further studies are needed to detect more chemical compositions and bioactivities of *R. rugosa* ‘Dianhong’ extracts affected by cultivars and the fermentation process.

## 4. Conclusions

The results of this study verified the importance of traditional knowledge in the fermentation of roses and demonstrated improvements in traditional fermentation techniques. In this study, fermented rose jams were prepared using *R. rugosa* ‘Dianhong’ petals with brown sugar, employing *S. rouxii* TFR-1 as a more effective strategy than natural fermentation. The results show that fermentation with *S. rouxii* TFR-1 significantly increased the total phenolic content, and antioxidant and SOD activities in YFR compared to NFR and the UFR (0 d). Additionally, *S. rouxii* TFR-1 fermentation shortens the fermentation period (around 7 d) to achieve optimum results compared to traditional natural fermentation. Furthermore, 15 phenolic compounds in NFR and YFR were identified and quantified using HPLC–MS/MS and HPLC–DAD methods. The study also examined how the content of these phenolic compounds changed with different fermentation techniques and durations. Moreover, the trends in phenolic compounds during the fermentation of *R. rugosa* ‘Dianhong’ were similar to those observed in the DPPH, ABTS^+^, FRAP, SOD, and TPC values, suggesting that these compounds significantly contribute to the antioxidant activity of fermented rose jams. Therefore, *R. rugosa* ‘Dianhong’ petals fermented with TFR-1 for 7 d represent a highly efficient product with high phenolic content and strong antioxidant activity. This provides a foundation for further studies on the compositions and bioactivities of different cultivars in the *Rosa* genus fermented with TFR-1 or other strains over a period of two weeks. Hence, this study could potentially promote the standardized production and commercialization of fermented *R. rugosa* ‘Dianhong’, offering broad applications in the food, cosmetics, and healthcare industries.

## Figures and Tables

**Table 1:** The analysis of phenolic compositions.

No.	Compounds	Rt. (min)	Ion Mode	MS/MS (m/s)	Contents (μg/mL)		
					UFR (0 d)	NFR (14 d)	YFR (7 d)
1	Gallic acid	0.74	Negative	169.0/124.9	27.93 ± 0.86 c	35.56 ± 2.03 b	48.25 ± 2.53 a
2	Protocatechuic acid	1.07	Negative	153.0/108.9	10.73 ± 0.56 a	4.96 ± 0.33 b	10.11 ± 0.68 a
3	P-hydroxybenzoic acid	1.65	Positive	138.9/95.0	0.10 ± 0.03 a	1.06 ± 0.14 b	1.05 ± 0.10 b
4	Vanillic acid	2.00	Positive	169.0/93.0	0.84 ± 0.12 c	1.58 ± 0.18 b	2.28 ± 0.30 a
5	Syringic acid	2.27	Positive	199.1/140.1	2.95 ± 0.34 b	2.62 ± 0.29 b	3.98 ± 0.47 a
6	Rutin	3.94	Positive	611.3/303.0	3.45 ± 0.52 a	3.61 ± 0.36 a	3.95 ± 0.55 a
7	Quercitrin	4.19	Positive	449.1/303.0	131.90 ± 7.30 b	152.90 ± 7.94 ab	175.02 ± 14.13 a
8	Quercetin	4.59	Positive	303.2/229.2	13.40 ± 1.30 b	61.95 ± 1.98 a	63.58 ± 2.56 a
9	Naringenin	4.68	Positive	273.1/153.1	0.08 ± 0.01 b	0.50 ± 0.04 a	0.56 ± 0.05 a
10	Kaempferol	4.85	Positive	287.1/153.1	2.46 ± 0.25 b	17.79 ± 0.97 a	17.84 ± 1.24 a
11	Apigenin	4.83	Positive	271.2/153.1	0.02 ± 0.00 c	0.06 ± 0.00 b	0.11 ± 0.05 a
12	Luteolin	4.63	Positive	287.0/153.0	1.21 ± 0.14 b	0.97 ± 0.05 b	1.58 ± 0.16 a
13	Chlorogenic acid	1.86	Positive	355.4/163.0	1.25 ± 0.11 a	0.43 ± 0.03 c	0.94 ± 0.12 b
14	Ferulic acid	3.73	Positive	195.1/177.0	0.13 ± 0.02 b	0.15 ± 0.01 b	0.20 ± 0.02 a
15	Catechin	1.37	Positive	291.0/139.1	0.23 ± 0.03 a	<0	0.09 ± 0.01 ab
16	In total	-	-	-	195.70 ± 11.58 c	284.31 ± 14.34 b	329.54 ± 18.01 a

Notes: Values are presented as mean ± standard deviation (n = 3) and evaluated by one-way ANOVA (post hoc test: Duncan test). Different lowercase letters (a–c) for the same compound represent significant differences (*p* < 0.05), while the same lowercase letters (a–c) for the same compound represent no significant difference (*p* > 0.05).

## Data Availability

Data are contained within the article.

## References

[1] Wang F., Miao M., Xia H., Yang L.G., Wang S.K. (2017). Antioxidant activities of aqueous extracts from 12 Chinese edible flowers in vitro and in vivo. Food Nutr. Res..

[2] Ren G.X., Xue P., Sun X.Y., Zhao G. (2018). Determination of the volatile and polyphenol constituents and the antimicrobial, antioxidant, and tyrosinase inhibitory activities of the bioactive compounds from the by-product of *Rosa rugosa* Thunb. Var. plena Regal tea. BMC Complement Altern. Med..

[3] Liu L., Tang D., Zhao H., Xin X., Aisa H.A. (2017). Hypoglycemic effect of the polyphenols rich extract from *Rosa rugosa* Thunb on high fat diet and STZ induced diabetic rats. J. Ethnopharmacol..

[4] Nađpal J.D., Lesjak M.M., Mrkonjić Z.O., Majkić T.M., Četojević-Simin D.D., Mimica-Dukić N.M., Beara I.N. (2018). Phytochemical composition and in vitro functional properties of three wild rose hips and their traditional preserves. Food Chem..

[5] Jiménez S., Gascón S., Luquin A., Laguna M., Ancin-Azpilicueta C., Rodriguez-Yoldi M.J. (2016). *Rosa canina* extracts have antiproliferative and antioxidant effects on Caco-2 human colon cancer. PLoS ONE.

[6] Liaudanskas M., Noreikienė I., Zymonė K., Juodytė R., Žvikas V., Janulis V. (2021). Composition and Antioxidant Activity of Phenolic Compounds in Fruit of the Genus *Rosa* L.. Antioxidants.

[7] Li M.X., Xie J., Bai X., Du Z.Z. (2021). Anti-aging potential, anti-tyrosinase and antibacterial activities of extracts and compounds isolated from *Rosa chinensis* cv. ‘JinBian’. Ind. Crop. Prod..

[8] Shameh S., Hosseini B., Alirezalu A., Maleki R.A. (2018). *Rosa* Phytochemical Composition and Antioxidant Activity of Petals of Six Species from Iran. J. AOAC Int..

[9] Xiong L.N., Yang J.J., Jiang Y.R. (2014). Phenolic compounds and antioxidant capacities of 10 common edible flowers from China. Food Sci..

[10] Lee M.H., Nam T.G., Lee I. (2018). Skin anti-inflammatory activity of rose petal extract (*Rosa gallica*) through reduction of MAPK signaling pathway. Food Sci. Nutr..

[11] Ju S.E., Ah-Ram H., Lee M.H. (2019). Extraction conditions for *Rosa gallica* petal extracts with anti-skin aging activities. Food Sci. Biotechnol..

[12] Wang Z., Wang Q., Tang K., Zhang H., Yang J., Qiu X., Jian H., Du G., Yan H. (2019). Analysis of floral scent composition and expression of key floral scent genes of Yunnan main grown edible roses. Plant Physiol..

[13] Wei L., Li J., Yang Y., Zhu M., Zhao M., Yang J., Yang Z., Zhou L., Zhou S., Gong J. (2022). Characterization and potential bioactivity of polyphenols of *Rosa rugosa*. Food Biosci..

[14] Wang Y., Zhao Y., Liu X., Li J., Zhang J., Liu D. (2022). Chemical constituents and pharmacological activities of medicinal plants from *Rosa* genus. Chin. Herb. Med..

[15] Xia A.-N., Liu L.-X., Tang X.-J., Lei S.-M., Meng X.-S., Liu Y.-G. (2022). Dynamics of microbial communities, physicochemical factors and flavor in rose jam during fermentation. LWT.

[16] Cendrowski A., Ścibisz I., Kieliszek M., Kolniak-Ostek J., Mitek M. (2017). UPLC-PDA-Q/TOF-MS Profile of Polyphenolic Compounds of Liqueurs from Rose Petals (*Rosa rugosa*). Molecules.

[17] Wang G.H., Lin Y.M., Kuo J.T. (2019). Comparison of biofunctional activity of *Asparagus cochinchinensis* (Lour.) Merr. Extract before and after fermentation with *Aspergillus oryzae*. J. Biosci Bioeng..

[18] Leonardo S., Jesús M.C., Paola M.R., Alejandro Z.C., Juan A.V., Cristóbal Noé A. (2020). Solid-state fermentation with *Aspergillus niger* GH1 to enhance polyphenolic content and antioxidative activity of Castilla Rose (*Purshia plicata*). Plants.

[19] Hur S.J., Lee S.Y., Kim Y.C. (2014). Effect of fermentation on the antioxidant activity in plant-based foods. Food Chem..

[20] Hu Y., Wang X.Y., Qin C.Q. (2022). Fermentation of rose residue by *Lactiplantibacillus plantarum* B7 and *Bacillus subtilis* natto promotes polyphenol content and beneficial bioactivity. J. Biosci. Bioeng..

[21] Zhou D.D., Saimaiti A., Luo M., Huang S.Y., Xiong R.G., Shang A., Gan R.Y., Li H.B. (2022). Fermentation with tea residues enhances antioxidant activities and polyphenol contents in kombucha beverages. Antioxidants.

[22] Zhao J., Yu J., Zhi Q., Yuan T., Lei X., Zeng K., Ming J. (2021). Anti-aging effects of the fermented anthocyanin extracts of purple sweet potato on *Caenorhabditis elegans*. Food Func..

[23] Cai Y.Y., Merhaba A., Gao L., Yang L.X. (2024). Analysis of Phenolic Content and its Antioxidant and Anti-inflammatory Activities during the Fermentation Process of *Rosa rugosa ‘*Dianhong’. Sci. Technol. Food Ind..

[24] Dżugan M., Tomczyk M., Sowa P., Grabek-Lejko D. (2018). Antioxidant activity as biomarker of honey variety. Molecules.

[25] Chen F., Huang G., Yang Z., Hou Y. (2019). Antioxidant activity of *Momordica charantia* polysaccharide and its derivatives. Intl. J. Biol. Macromol..

[26] Deseo M.A., Elkins A., Rochfort S. (2020). Antioxidant activity and polyphenol composition of sugarcane molasses extract. Food Chem..

[27] Gu C., Howell K., Dunshea F.R., Suleria H.A. (2019). LC-ESI-QTOF/MS haracterization of phenolic acids and flavonoids in polyphenol-rich fruits and vegetables and their potential antioxidant activities. Antioxidants.

[28] Dai H., Jiang B., Zhao J., Li J., Sun Q. (2022). Metabolomics and Transcriptomics Analysis of Pollen Germination Response to Low-Temperature in Pitaya (*Hylocereus polyrhizus*). Front. Plant Sci..

[29] Abdellatif F., Begaa S., Messaoudi M., Benarfa A., Ouakouak H., Hassani A., Sawicka B., Simal Gandara J. (2023). HPLC–DAD analysis, antimicrobial and antioxidant properties of aromatic herb *Melissa officinalis* L.; aerial parts extracts. Food Anal. Methods.

[30] Xiong R.G., Wu S.X., Cheng J., Saimaiti A., Liu Q., Shang A., Zhou D.D., Huang S.Y., Gan R.Y., Li H.B. (2023). Antioxidant Activities, Phenolic Compounds, and Sensory Acceptability of Kombucha-Fermented Beverages from Bamboo Leaf and Mulberry Leaf. Antioxidants.

[31] Zhao C.N., Tang G.Y., Cao S.Y., Xu X.Y., Gan R.Y., Liu Q., Mao Q.Q., Shang A., Li H.B. (2019). Phenolic profiles and antioxidant activities of 30 tea infusions from green, black, oolong, white, yellow and dark teas. Antioxidants.

[32] Xia A.-N., Meng X.-S., Tang X.-J., Zhang Y.-Z., Lei S.-M., Liu Y.-G. (2022). Probiotic and related properties of a novel lactic acid bacteria strain isolated from fermented rose jam. LWT.

[33] Sharma R., Garg P., Kumar P., Bhatia S.K., Kulshrestha S. (2020). Microbial Fermentation and Its Role in Quality Improvement of Fermented Foods. Fermentation.

[34] Lin M.Y., Yen C.L. (1999). Antioxidative ability of lactic acid bacteria. J. Agric. Food Chem..

[35] Espirito-Santo A.P., Carlin F., Renard C.M. (2015). Apple, grape or orange juice: Which one offers the best substrate for lactobacilli growth?—A screening study on bacteria viability, superoxide dismutase activity, folates production and hedonic characteristics. Food Res. Int..

[36] Zhang M.W., Zhang R.F., Zhang F.X., Liu R.H. (2010). Phenolic profiles and antioxidant activity of black rice bran of different commercially available varieties. J. Agric. Food Chem..

[37] Nguela J.M., Vernhet A., Julien-Ortiz A., Sieczkowski N., Mouret J.R. (2019). Effect of grape must polyphenols on yeast metabolism during alcoholic fermentation. Food Res. Int..

[38] Minnaar P.P., Jolly N.P., Paulsen V., Du Plessis H.W., Van Der Rijst M. (2017). *Schizosaccharomyces pombe* and *Saccharomyces cerevisiae* yeasts in sequential fermentations: Effect on phenolic acids of fermented Kei-apple (*Dovyalis caffra* L.) juice. Intl. J. Food Microbiol..

[39] Sang S., Lapsley K., Jeong W.S., Lachance P.A., Ho C.T., Rosen R.T. (2002). Antioxidative phenolic compounds isolated from almond skins (*Prunus amygdalus* Batsch). J. Agric. Food Chem..

[40] Palafox-Carlos H., Yahia E.M., González-Aguilar G.A. (2012). Identification and quantification of major phenolic compounds from mango (*Mangifera indica*, cv. Ataulfo) fruit by HPLC–DAD–MS/MS-ESI and their individual contribution to the antioxidant activity during ripening. Food Chem..

[41] Zhang H., Liu Y.-Z., Xu W.-C., Chen W.-J., Wu S., Huang Y.-Y. (2020). Metabolite and Microbiome Profilings of Pickled Tea Elucidate the Role of Anaerobic Fermentation in Promoting High Levels of Gallic Acid Accumulation. J Agric. Food Chem..

[42] Fernandez-Orozco R., Frias J., Muñoz R., Zielinski H., Piskula M.K., Kozlowska H., Vidal-Valverde C. (2007). Fermentation as a Bio-Process To Obtain Functional Soybean Flours. J. Agric. Food Chem..

[43] Kim S.S., Park K.J., An H.J., Choi Y.H. (2017). Phytochemical, antioxidant, and antibacterial activities of fermented *Citrus unshiu* byproduct. Food Sci. Biotechnol..

[44] Al-Yafeai A., Bellstedt P., Böhm V. (2018). Bioactive Compounds and Antioxidant Capacity of *Rosa rugosa* Depending on Degree of Ripeness. Antioxidants.

